# Systematic computational hunting for small RNAs derived from ncRNAs during dengue virus infection in endothelial HMEC-1 cells

**DOI:** 10.3389/fbinf.2024.1293412

**Published:** 2024-01-31

**Authors:** Aimer Gutierrez-Diaz, Steve Hoffmann, Juan Carlos Gallego-Gómez, Clara Isabel Bermudez-Santana

**Affiliations:** ^1^ Grupo Rnomica Teórica y Computacional, Departamento de Biología, Facultad de Ciencias, Universidad Nacional de Colombia, Bogotá, Colombia; ^2^ Faculty of Biosciences, Leibniz Institute on Aging—Fritz Lipmann Institute (FLI), Friedrich Schiller University Jena, Jena, Germany; ^3^ Molecular and Translational Medicine Group, Medicine Faculty Universidad de Antioquia, Medellin, Colombia

**Keywords:** differential expression, small RNASeq, ncRNAs, multimapping problem, dengue virus, tRNA-derived fragments

## Abstract

In recent years, a population of small RNA fragments derived from non-coding RNAs (sfd-RNAs) has gained significant interest due to its functional and structural resemblance to miRNAs, adding another level of complexity to our comprehension of small-RNA-mediated gene regulation. Despite this, scientists need more tools to test the differential expression of sfd-RNAs since the current methods to detect miRNAs may not be directly applied to them. The primary reasons are the lack of accurate small RNA and ncRNA annotation, the multi-mapping read (MMR) placement, and the multicopy nature of ncRNAs in the human genome. To solve these issues, a methodology that allows the detection of differentially expressed sfd-RNAs, including canonical miRNAs, by using an integrated copy-number-corrected ncRNA annotation was implemented. This approach was coupled with sixteen different computational strategies composed of combinations of four aligners and four normalization methods to provide a rank-order of prediction for each differentially expressed sfd-RNA. By systematically addressing the three main problems, we could detect differentially expressed miRNAs and sfd-RNAs in dengue virus-infected human dermal microvascular endothelial cells. Although more biological evaluations are required, two molecular targets of the hsa-mir-103a and hsa-mir-494 (CDK5 and PI3/AKT) appear relevant for dengue virus (DENV) infections. Here, we performed a comprehensive annotation and differential expression analysis, which can be applied in other studies addressing the role of small fragment RNA populations derived from ncRNAs in virus infection.

## Introduction

Non-coding RNAs (ncRNAs) are functional RNA molecules involved in many cellular processes. During the last 20 years, ncRNAs have repeatedly been reported as sources of small RNAs. For example, alternative RNA processing steps for tRNAs give rise to smaller functional RNAs (Cole et al., 2009; Lee et al., 2009; Lyons et al., 2018). Specifically, studies have found small RNA fragments between 20 and 30 nt in length mainly derived from tRNAs with a functional resemblance to the Argonaute–miRNA gene silencing pathway (Cole et al., 2009; Kumar et al., 2014; Kuscu et al., 2018). These processes can be observed across the tree of life. For instance, specific cleavage and processing of tRNAs have also been observed in the fungus *Aspergillus fumigatus* (Christoph et al., 2008) or human small RNA-seq libraries (Kawaji et al., 2008). In addition, recent surveys have also recognized that viruses employ a mechanism to suppress antiviral responses that involve the expression of RNA fragments derived from the host transfer RNA, which were in the past the so-called tRNA-derived fragments, or tRFs (Ivanov, 2015). For instance, respiratory syncytial virus (RSV)-induced tRFs derived from the 5-end of mature tRNAs decoding GlyCCC, LysCTT, and CysGCA (named tRF5-GlyCCC, tRF5-LysCTT, and tRF5-CysGCA, respectively) (Zhou et al., 2017) can target specific genes, e.g., SYNE-2, MIP-1*β*, and IP-10 (Chen and Bao, 2020). tRFs have also been associated with antibody response to bovine leukemia virus (BLV) in Holstein cattle (Taxis et al., 2018). Finally, regulating La/SSB-dependent viral gene expression by pre-tRNA 3’ trailer-derived small RNAs has been experimentally tested under various RNA virus infections (Cho et al., 2019). Apart from tRNAs, other ncRNAs linked to small RNA expression are precursor–microRNA (pre-miRNA) hairpins that give rise to additional micro RNA offsetRNAs (moRNAs) in *Ciona intestinalis* (Shi et al., 2009) and in the human brain (Langenberger et al., 2009). Small nucleolar RNAs (snoRNAs) have also been reported as a source for specific miRNA-like short RNAs (Ender et al., 2008; Saraiya and Wang, 2008; Taft et al., 2009) as well as for the reported data for vault RNAs (Persson et al., 2009; Stadler et al., 2009).

Overall, small RNA-mediated gene regulation may be even more complicated than previously believed (Christoph et al., 2008), e.g., affecting the human brain (Kawaji et al., 2008) or potentially influencing human diseases (Rashad et al., 2020). Therefore, the detection of new small RNA *loci* derived from annotated or un-annotated ncRNAs is a relevant problem to many fields in life sciences, in particular for inferring new ncRNA–disease connections, e.g., in cancer research (Zhu et al., 2020). Though next-generation sequencing (NGS) protocols have enabled us to discover expression profiles for many coding genes, those approaches may need to be more straightforwardly applied to ncRNAs. Therefore, to comprehensively study the expression profile of ncRNAs, several requirements must be addressed to minimize, for example, the impact of data analysis methods on the recovery of the accuracy of miRNA profiles (Tam et al., 2015). A more fine-grained transcriptome data analysis to identify alternative small RNA processing patterns could provide the opportunity to detect new biological pathways for small RNA processing and its associated functions. However, to accurately detect sfd-RNAs, it is necessary to improve the accuracy of detection of small RNA profiles.

This study addressed the problems relevant to small RNA detection derived from NGS data preprocessing and ncRNA annotations that come partly from the ncRNA copy-number expansions in genome evolution. Accurate annotation of ncRNAs and the effect of the multi-mapping read (MMR) placement problem on differential expression analysis. We tackle the problem of assigning the correct expression levels of ncRNAs because their expansion during genome evolution lets them be presented as multicopy genes, as seen in the case of tRNAs in many eukaryotes (Bermudez-Santana et al., 2010; Velandia-Huerto et al., 2016). In addition, other kinds of repeated and non-coding regions exist in eukaryotic chromosomes—centromeres, pericentromeric regions, and telomeric regions— and are interspersed between them. However, establishing their function is currently being researched since authors recognize the need to improve methods to study the biology and methodology of small RNA in wet-lab experiments and silico approaches. However, new advances are found in the study by Louzada et al. (2020) and Lopes et al. (2023).

We developed a solution to these problems by detecting several differentially expressed sfd-RNAs that might activate in the host by DENV infection. It remains to be evaluated if they have a unique role in non-canonical RNAi pathways. Nonetheless, during the last decade, the importance of ncRNAs in the regulation of viral replication, viral persistence, host immune evasion, and cellular transformation has increasingly gained attention, e.g., in the case of viral non-coding RNAs (Tycowski et al., 2015; Ivanov, 2015; Nunes et al., 2020). Recent results from the study by our group suggest a potential biological role for some sfd-RNA candidates. For some miRNAs on the starting point, consolidation, and finishing of the viral cycle of DENV in cell cultures (Roa-Linares, 2019; Cuartas-Lopez et al., 2018), assessing the biological function for the majority of the sfd-RNAs detected here remains a future challenge.

## Results

### ncRNA unified annotation, without copy-number limitations

The annotation of families of miRNAs, tRNAs, and snoRNAs in public databases remains problematic (Kozomara and Griffiths-Jones, 2013; Telonis et al., 2015; Jorjani et al., 2016) and creates ambiguities in the assignment of sfd-RNAs to their sources (Schopman et al., 2010). In addition, the multicopy nature of ncRNAs aggravates the problem of assigning sources for sfd-RNAs and establishing their new biological function. However, few *loci* showed strong evidence for a natural role (Hoeppner et al., 2018).

Despite experimental constraints to detecting and testing the expression for ncRNAs in multiple copies, we faced the problem of providing a measure of specificity for an ncRNA to be able to code for specific small RNAs. For instance, categories of homologous ncRNA sequences were defined to solve the conflict of unsolved annotation for ncRNAs. This approach also helped clean identifiers approved by the HGCN. We set some categories to help simultaneously classify and integrate ncRNA annotation and avoid species-specific annotation changes due to the curation; for instance, we used ncRNA annotation of conserved sequences in primates. Additionally, we included all ncRNA categories to classify and integrate the ncRNAs even in cases in which an ncRNA fit multiple class annotations as was seen for the *loci* coding the hsa-mir-768 and the snoRNA HBII-239 (Hüttenhofer et al., 2001). Furthermore, a method to reduce the overrepresented identical paralogs of ncRNAs was included (see methods: *Consensus of multicopies of ncRNA loci*). Our strategy to solve the conflicting annotations and the integration of the ncRNA gene features allowed us to find several sfd-RNAs annotated under other ncRNA categories, such as piRNAs and snoRNAs. Notoriously, those piRNAs resemble the coordinates of recent significant discoveries such as the piR-hsa-23289, which have the same genome coordinates defined for the tRF experimental validated tRF-5′-GluCTC (Wang et al., 2013).

### Effect of multi-mapping reads on ncRNAs

**FIGURE 1:**
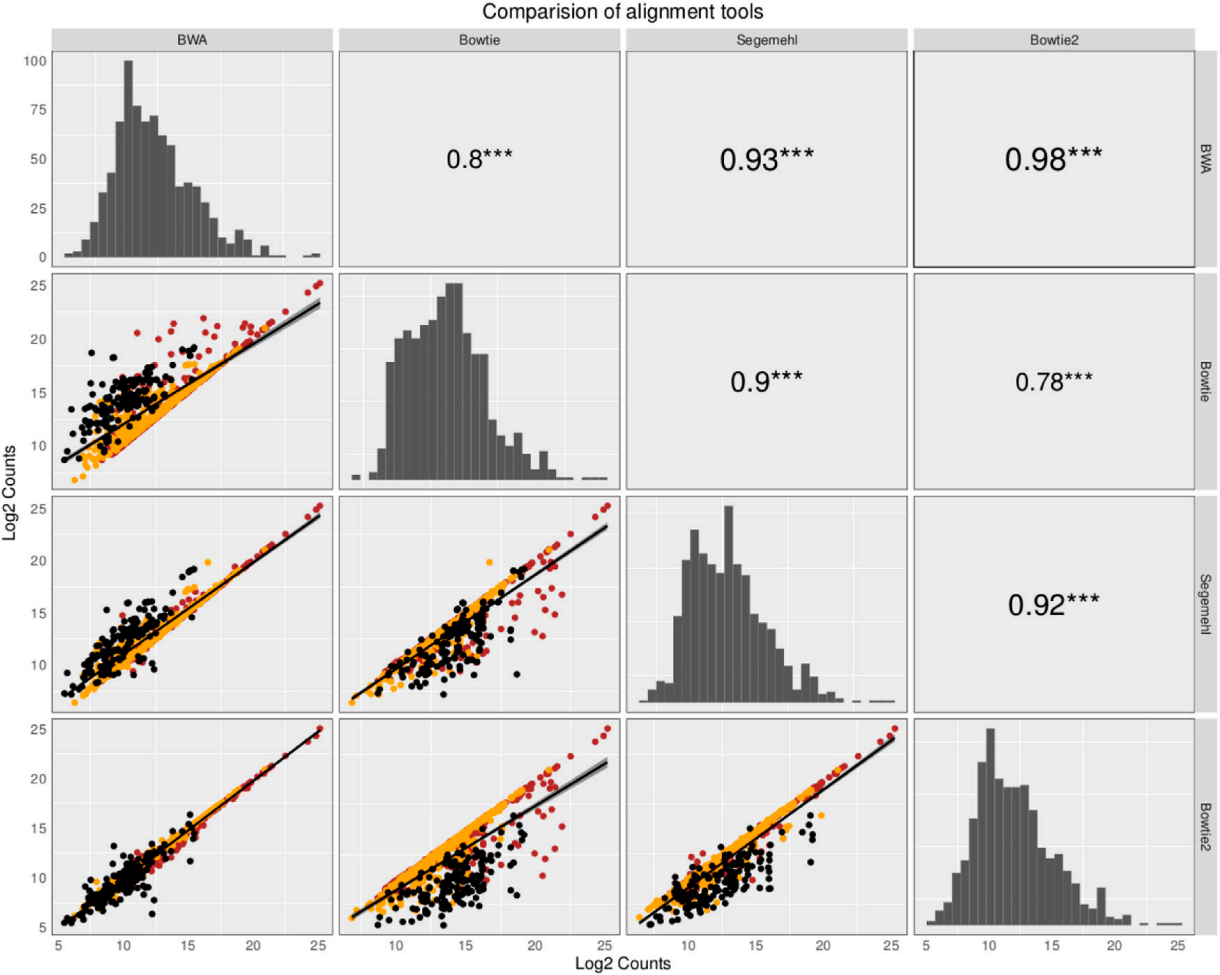
Comparison of the abundance and expression of 324 ncRNA loci with reads commonly mapped by the four aligners. The plots compare the four aligners both in the columns and rows. This arrangement leaves each method to cross itself on time, with the diagonal showing the read distribution for each aligner. Each method crosses the other two times as well. In this sense, the first intersection will portray the spot distribution (reads mapped to each ncRNA) to calculate Spearman’s correlation, the value of which is displayed on the second intersection (upper right corner). Reads commonly mapped by all the different aligners were averaged across the samples and plotted for pairwise comparisons using Spearman’s correlation, as suggested in Tam et al. (2015). The ncRNAs are colored based on class: tRNAs in black, snoRNAs in orange, and miRNAs in red. The read distribution for each aligner is shown on the diagonal. Spearman’s correlation is depicted with the significance in the upper right corner of the pairwise comparisons. In the bottom left corner, the log2 counts of each ncRNA are shown.

**FIGURE 2:**
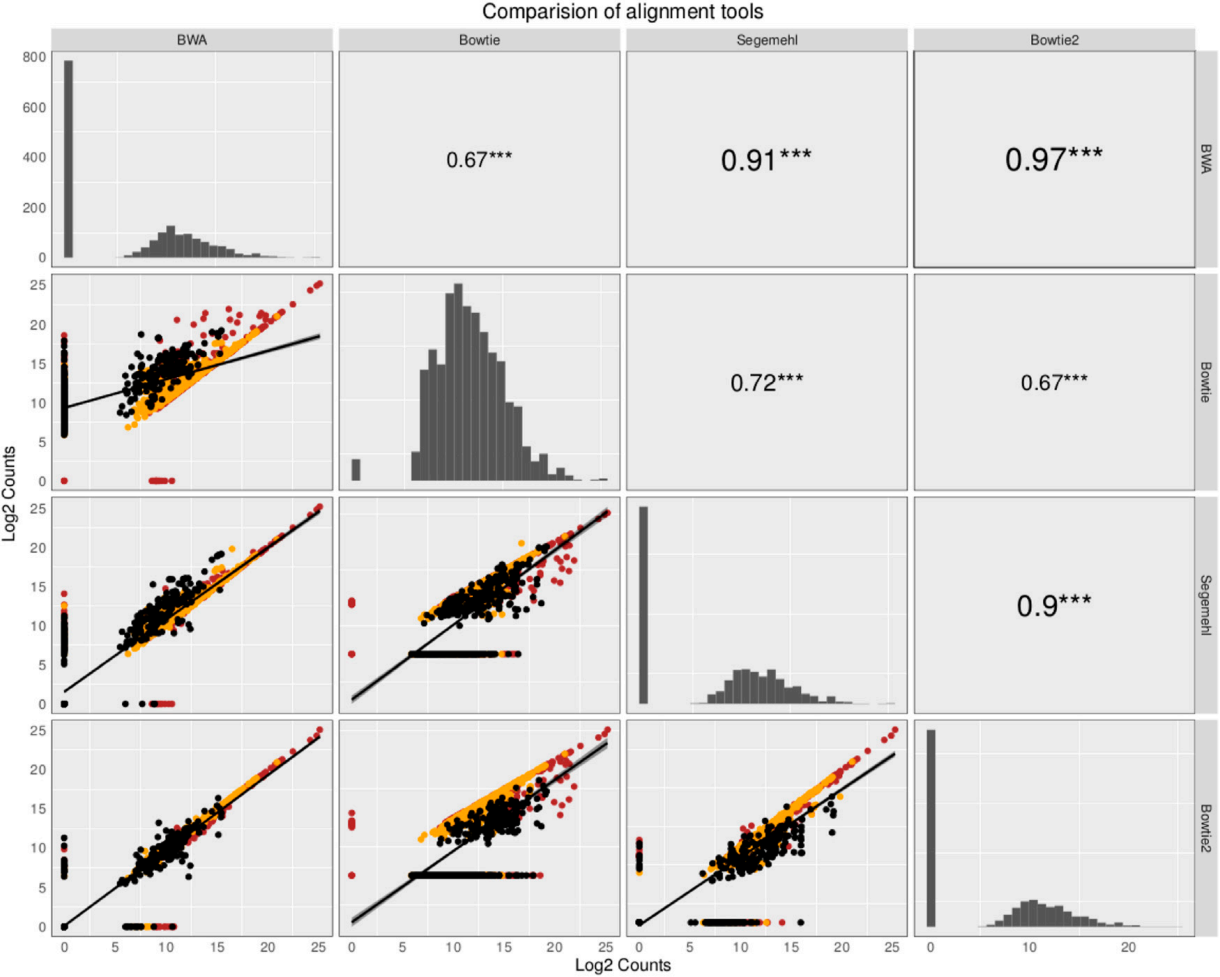
Comparison of the abundance and expression of the total number of ncRNA with read alignments. The plots compare the four aligners both in the columns and rows. This arrangement leaves each method to cross itself on time, with the diagonal showing the read distribution for each aligner. Each method crosses the other two times as well. In this sense, the first intersection will portray the spot distribution (reads mapped to each ncRNA) to calculate Spearman’s correlation, the value of which is displayed on the second intersection (upper right corner). The ncRNAs are colored based on class: tRNAs in black, snoRNAs in orange, and miRNAs in red. Here, all the mapped reads are included, regardless of whether all the aligners commonly map the reads. Although some aligners have a high correlation value, due to the similarities in the alignment algorithm, each mapper produces a new particular output, giving evidence that the aligner methods influence the expected results in research of small ncRNAs.

The small RNA sequencing (RNA-seq) products analyzed here are derived from two fractions of RNA of sizes ranging from 15 to 30 nt (small-sized fraction) and from 30 to 200 nt (medium-sized fraction). Fractions were size-purified from total RNA isolated from DENV2-infected endothelial (HMEC-1) cells and non-infected HMEC-1 cells (mock cells–controls) at different time points. Reads were aligned to 4,320 ncRNAs by all four aligners. Then, two comparisons were made to identify the bias generated by each aligner method. Those results have been portrayed in Figures 1, 2. The plots compare the four aligners, both in columns and rows. This arrangement leaves each method to cross itself on time, with the diagonal showing the read distribution for each aligner. Each method crosses the other two times as well. In this sense, the first intersection will portray the spot distribution (reads mapped to each ncRNA) to calculate Spearman’s correlation, the value of which is displayed on the second intersection (upper right corner).

In the first comparison, reads commonly mapped by all the different aligners were averaged across the samples and plotted for pairwise comparisons using Spearman’s correlation as suggested in Tam et al. (2015). In Figure 1, which contains information from that comparison, the value of Spearman’s correlation coefficient is over 0.9 for all the pairwise comparisons, except for the comparison with Bowtie, but the correlation between Bowtie and segemehl is 0.9, which can handle MMR reads and recover the highest number of reads aligned to ncRNAs. However, the restriction to working only with reads mapped by all the aligners reduces the specific advantages that each mapping algorithm has. While BWA aligns MMRs with a random behavior to one genomic location, Bowtie, Bowtie2, and segemehl aligners generally avoid a pseudorandom distribution of reads among repetitions.

In contrast, in the second comparison, when all the ncRNAs (including all the mapped reads, regardless of whether all the aligners commonly map the reads) are included to compare the aligners, Spearman’s correlation behaves similarly between all the aligner values, but it changes dramatically in correlations mainly in paired comparisons between other aligners and Bowtie (Spearman’s correlation coefficient varies from 0.67 to 0.72).; see the behavior in Figure 2. Interestingly, several ncRNAs explicitly detected by this aligner were also detected as differentially expressed (see the following section). Intriguingly, noticing how the read distribution changes for each aligner on the diagonal arrangement is essential. Furthermore, we noted that ncRNAs with more copies, such as tRNAs, have an aligner-dependent behavior due to sfd-RNAs processed from these transcripts, mainly detected as differentially expressed by aligners with tolerance to MMRs.

### Differential expression detection of sfd-RNAs and miRNAs

**FIGURE 3:**
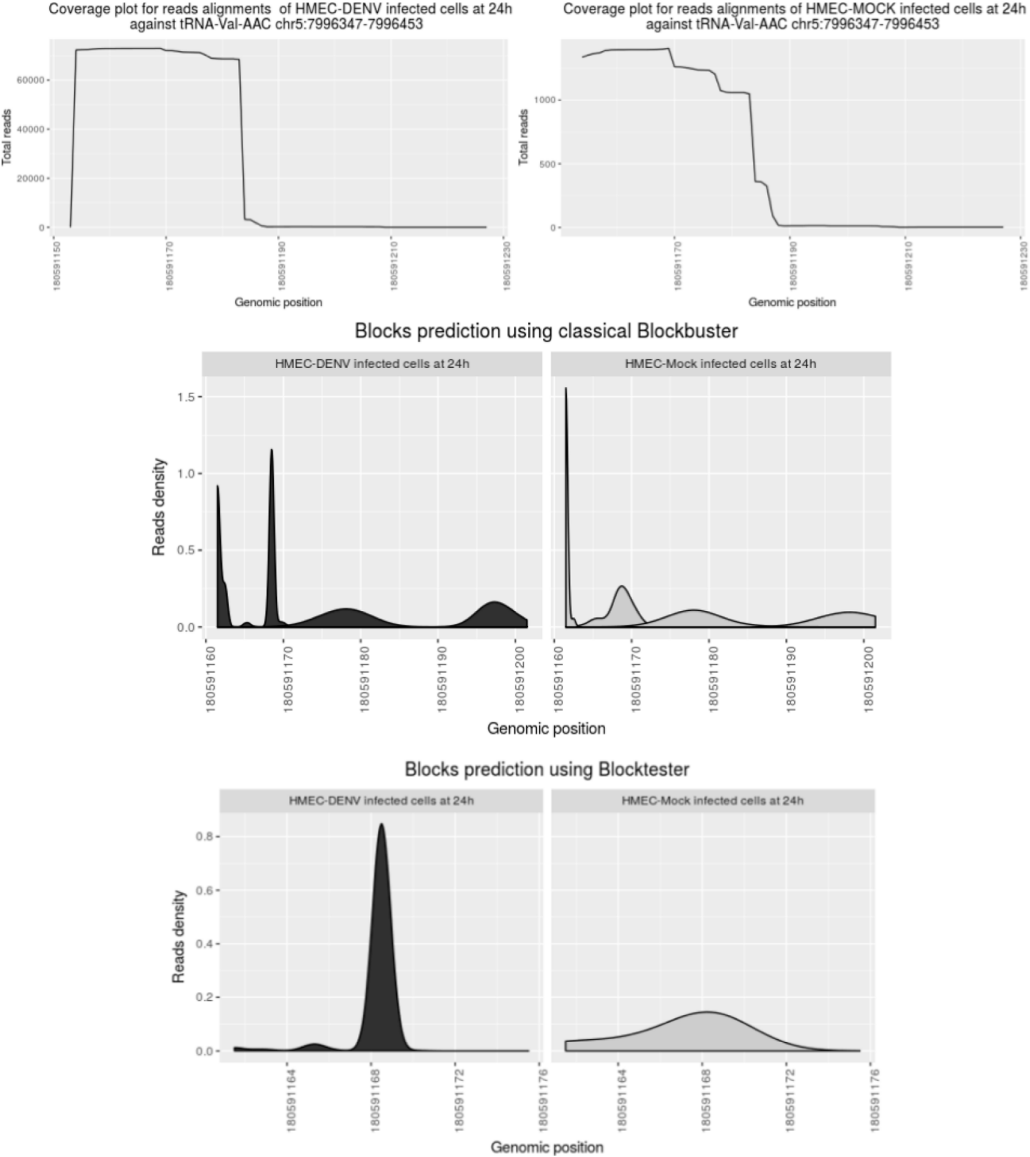
Comparison of block expression detection using the block tester on the locus tRNA Val-AAC. The profile of reads is converted to Gaussian distribution by Blockbuster. New blocks of expression and overlapped blocks are observed. After testing a block with a block tester, the sfd-RNA block at 5′tRNA is detected. Interestingly, the high selection between blocks of this sfd-RNA in infected and non-infected endothelial cells is quite different.

**FIGURE 4:**
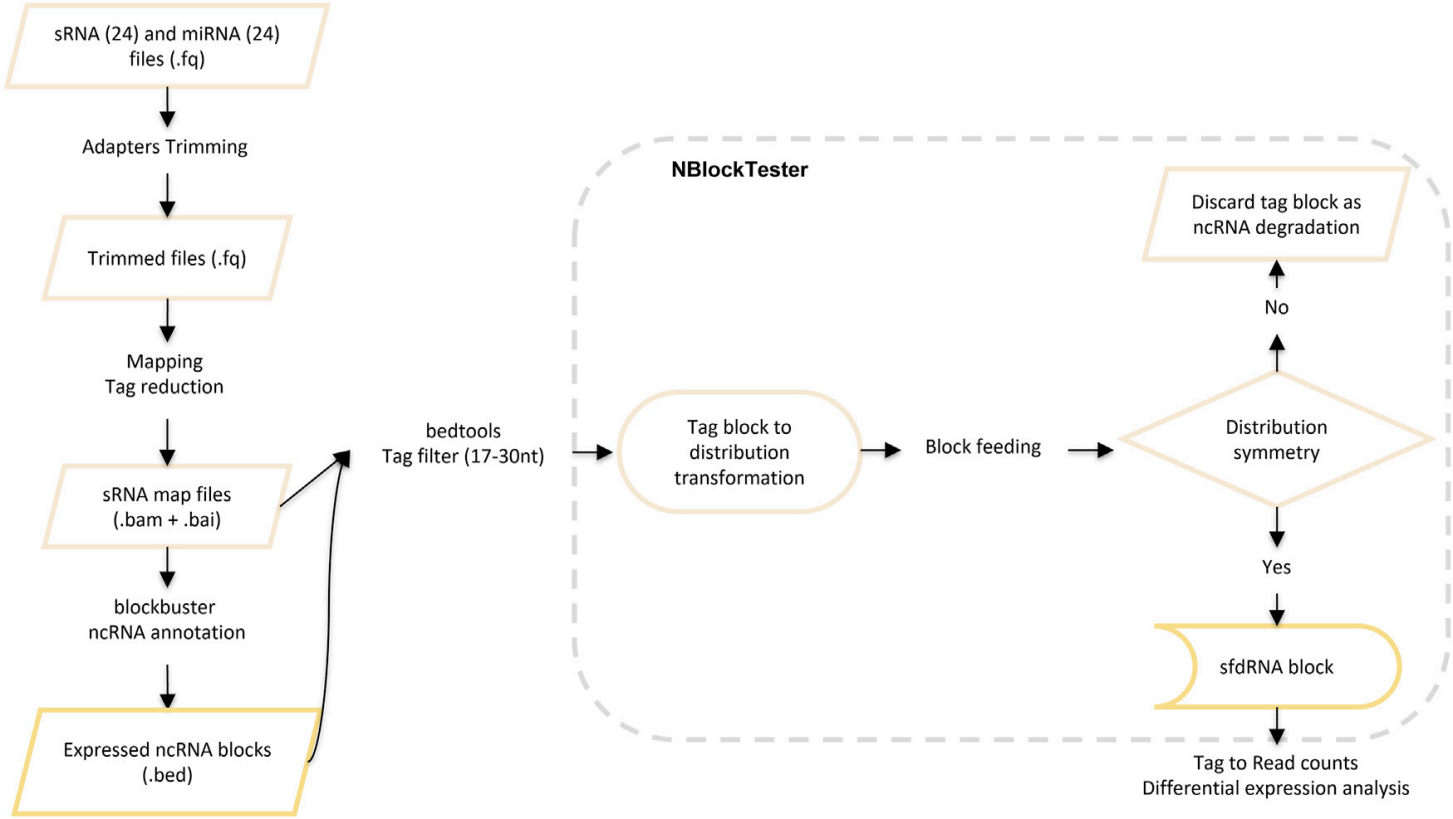
Description of the methodology for searching for expression patterns and fragment discrimination in blocks.

After mapping, we detected ncRNAs with complex profiles of expression. Those patterns exhibit nested blocks built of a pile of reads that arise from two different but adjacent positions in the same locus. Figure 3 illustrates a typical example showing such patterns. First, Blockbuster (Langenberger et al., 2009) was used to detect the global expression and patterns for each ncRNA locus once the ncRNA *loci* or classes were defined. Then, a further step was carried out to tune the detection of expression profiles of sfd-RNAs embedded in a complex distribution of reads. In this new step, a test over each block of the entire mapped region is done independently of the accumulation of dispersed reads (see methods: *Searching for expression patterns and fragment discrimination in blocks. See Figure 4 for the summary of the explanation of the procedure*). This new step uncovered several small RNAs on *loci*, which had not previously been associated with production of small RNAs. Then, small- and medium-sized fractions were contrasted to discriminate between degradation fragments or non-fragmented ncRNAs and induced sfd-RNAs by dengue virus infection. After comparing and measuring the basal expression of ncRNAs in uninfected (mock cells–controls) and infected cells in the medium-sized fraction, the small-sized fraction was used to detect the differential expression by contrasting block expressions against the controls simultaneously in both fractions. Putative sfd-RNAs in blocks were deemed differentially expressed if the expression level changes were statistically significant under dengue virus infection at any time post-infection, but none in any control.

**FIGURE 5:**
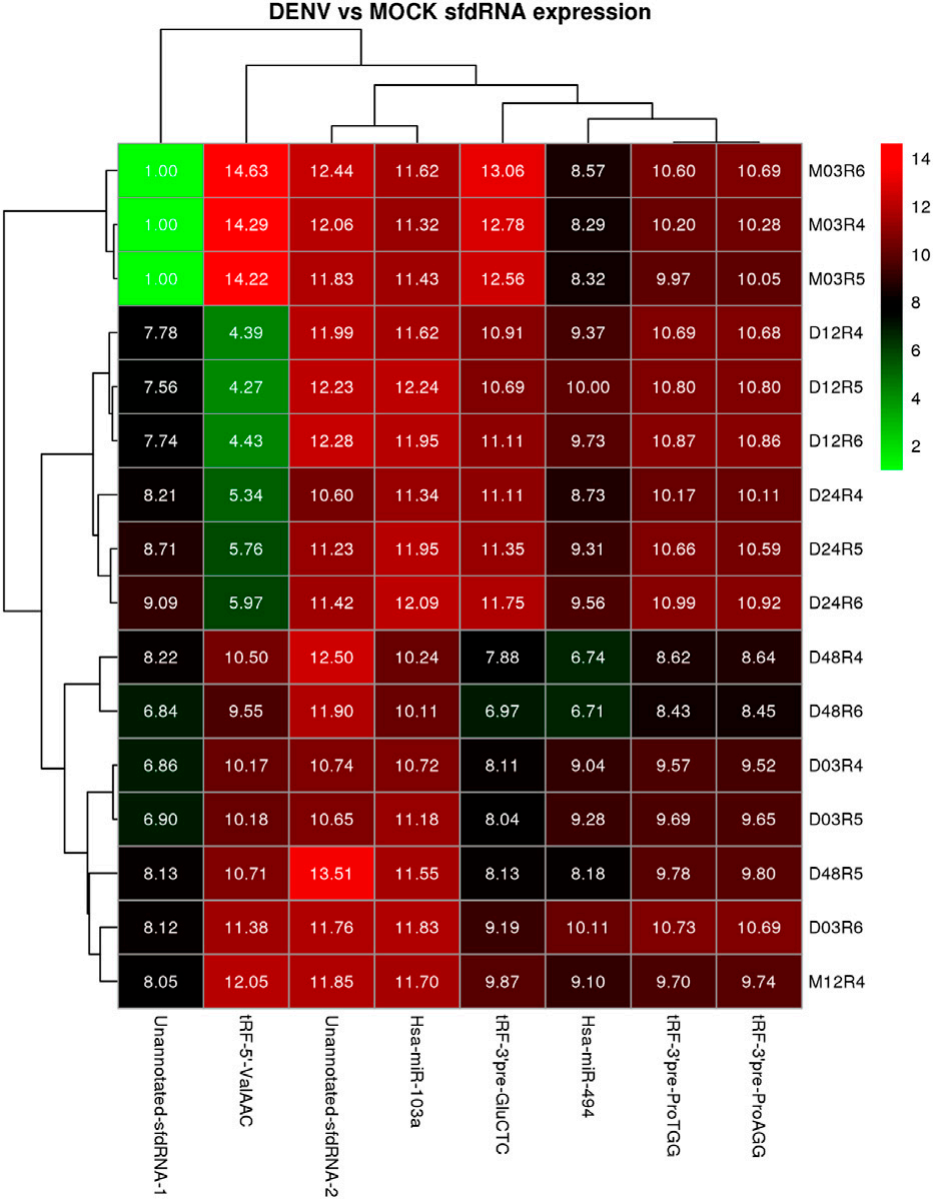
Heatmap and cluster patterns of the differentially expressed ncRNAs among samples. Samples exhibiting similar ncRNA expression values are clustered together; notably, the DENV-infected samples at 3 and 24 h do not cluster with the mock control as is expected from the differential expression analysis.

The expression patterns for the selected differentially expressed sfd-RNAs are represented in a heatmap. The global profile for the candidates with the best statistical values and the best rank order of prediction for each differentially expressed sfd-RNA, shown in Figure 5. Apart from the miRNAs hsa-mir-103a and hsa-mir-494, tRNA-derived sfd-RNAs, e.g., tRF-3′ProTGG, tRF-3′ProAGG, tRF-3′ GluCTC, and tRF-5′ValACC, were also ranked with a consistent score of reproducibility of 1 for all the sixteen strategies, i.e., identified as differentially expressed by the four aligners and normalization methods. The summary of statistics of these results is shown in Table 1.

**TABLE 1:** Summary statistics of representative sfd-RNAs and miRNAs differentially expressed in DENV2-infected HMEC-1 cells. FC: fold change; CPM: count per million; LR: likelihood ratio statistics; FDR false discovery rate; Aligner and NM columns display the computational method where the sfdRNA has the highest LR value where Aligner represents the aligner tool and NM to the normalization method. RA is the fraction of reproducibility of the aligner concerning the other aligners, and RN is the fraction of reproducibility of all normalization methods. The abbreviations CPM, UQS, TMM, and RLE represent count per million, upper quartile scaling, trimmed Mean of M, and the relative log expression, respectively. The complete set of identified sfd-RNAs as DE is available in Supplementary Tables S1–S4.

sfd-RNA source	GRCh38.p14 position	hpi	logFC	logCPM	LR	*p*-value	FDR	Aligner	RA	NM	RN
POLA1	X:24970573–24970603	3	10.5	6.4	23.62	1.0E-6	8.0E-4	Bowtie2	0.5	UPQ	1
Not assigned	chr17:53088644–53088661	24	−3.16	10.51	12.58	4.0E-4	3.0E-2	Bowtie2	0.5	CPM	1
MIR103A2	chr20:3917541–3917563	48	4.33	10.3	14.16	2.0E-4	2.0E-2	segemehl	0.25	UPQ	1
MIR494	chr14:101023375–101023396	48	6.21	7.92	15.16	1.0E-4	1.0E-2	segemehl	0.25	RLE	1
tRF-3′ tRNA-Pro-TGG-3-1	chr5:181188895–181188933	24	1.77	8.27	15.21	1.0E-4	1.0E-2	Bowtie2	0.25	RLE	1
tRF-3′tRNA-Pro-AGG-2-5	chr14:20609377–20609415	24	1.79	8.22	15.6	8.0E-5	1.0E-2	Bowtie2	0.25	RLE	1
tRF-3′tRNA-Glu-CTC-1-2 (pre-GluCTC)	chr1:161447258–161447307	48vs24	9.96	11.48	25.86	4,00E-07	0.0002	BWA	0.25	TMM	1
tRF-5′ TRV-AAC1-2 (tRNA-ValAAC)	chr5:181164154–181164183	48vs12	10.06	12.34	21.94	3.0E-6	3.0E-3	BWA	0.25	RLE	1

## Discussion

Interestingly, after dealing with the multi-mapped read problem and checking the mapping and normalization methods, we found intricate patterns of piles of mapped reads. As the first impression, those signals presumably indicate a pattern of reads that were generally considered random degradation products previously. While analyzing those intricate patterns, the automated detection of sfd-RNAs became another arduous task. Although tools like FlaiMapper (Hoogstrate et al., 2014) attempt to address the challenge of handling nested blocks, the problem is only sometimes solved. In short, because a minimum number of identical read starts and ends is required to annotate a block, piles of reads indicative of sfd-RNAs often need to be found. Our approach dealt with this problem, helped us solve complex read assignments, and facilitated differential expression analysis.

The proposed improvements help determine sets of up-and downregulated sfd-RNAs in particular experimental conditions. The expression analysis of hsa-mir-103a and hsa-mir-494 48 h after dengue virus infection is an essential example of this development. Nevertheless, tRFs were not always predicted simultaneously using the four aligners. Still, the advantage of using more than one strategy was highly desirable to detect them since a rank-order of prediction of 1 was observed for tRFs tRF-3′ProTGG, tRF-3′ProAGG, tRF-3′ GluCTC, and tRF-5′ValACC. Remarkably, tRF-5′ValACC got a fold change of 10.5 from 12 to 48 h post-infection. Process validation should be defined to document the evidence of this change.

The expression profile of the top eight differentially expressed sfd-RNAs in endothelial cells infected by DENV2 gave the best predictions for two miRNAs (hsa-mir-103a and hsa-mir-494) as differentially expressed between cell cultures. These results are compatible with those of our previous published work. Moncini et al. (2011) found out that the miR-103 is implied in regulating CDK5R1, a transcript related to cellular migration. In addition, Roa-Linares (2019) have reported that the loss of function of CDK5 causes cytoskeleton reorganization and a decrease in dengue virus infection. Additionally, miR-494 promotes PI3K/AKT pathway hyperactivation in human hepatocellular carcinoma progression (Lin et al., 2018). Recently, we found evidence from several experiments that PI3/AKT pathway activation controls the small RhoGTPases and ulterior remodeling of microfilaments of actin after dengue virus infection on hepatoma cell cultures (Cuartas-Lopez et al., 2018). In addition, the role of small RNAs in dengue virus 2-infected Aedes mosquito cells has also revealed viral piRNAs and novel host miRNA expression that changed substantially upon DENV2 infection (Miesen et al., 2016). In addition, sfd-RNAs derived from tRNAs, tRF-3′ProTGG, tRF-3′ProAGG, tRF-3′ GluCTC, and tRF-5′ValACC, were also ranked with a consistent score of reproducibility for all the sixteen strategies. These results are in agreement with the fact that for some tRFs, there is a substantial increase in their abundance in chronic viral infections of the liver caused by hepatitis B virus (HBV) or hepatitis C virus (HCV) (Selitsky et al., 2015). Similar tRFs expressed under other experimental conditions are deposited at the tRFdb (Kumar et al., 2015; tRFdb, 2020).

Our exhaustive analysis of the expression of small RNAs under DENV infection led us to detect candidates of sfd-RNAs related to other sfd-RNAs that were validated to be products of ncRNAs (Fort et al., 2022) or specifically of tRNAs (Cole et al., 2009) in viral infection conditions (Zhou et al., 2017; Taxis et al., 2018; Cho et al., 2019; Chen and Bao, 2020) or under other biological conditions that affect their cleavage and processing (Christoph et al., 2008; Kawaji et al., 2008; Hoogstrate et al., 2014)). Furthermore, our work proposes new candidates whose differential expression must be experimentally tested, particularly those suited to investigate whether our candidates indeed are regulated under dengue virus infection. This study has potential limitations. Our model is based on the annotation derived from hg19. They are, therefore, subject to biases and confounding factors that may have influenced our model estimates. However, the coordinates of differentially expressed sfd-RNA candidates were all corrected and assigned to the coordinates of the human GRCh38.p14 version with the accepted HGCN names of potential sources of sfd-RNAs (that information is available in Supplementary Tables S1–S4 including the coordinates of the sfd-RNAs, the nearest annotated source, and the distance to it).

## Conclusion

Our approach allowed us to quantify simultaneously the differential expression of miRNAs and sfd-RNAs by an exhaustive analysis of small RNA-seq data derived from DENV-2-infected endothelial cells. Spearman’s correlation coefficient value measured among the four types of aligners may suggest that the alignment method has a considerably more significant impact on the recovery of ncRNA abundance profiles than the library normalization method. The new ranking score introduced here helps prioritize subsequent experimental validation of candidate sfd-RNAs since it is based on the results of sixteen combinations of aligners and normalization methods. Our computational procedure allowed us to detect un-annotated *loci* acting as sources of small RNAs. We found significant ranking scores for the human miRNAs hsa-mir-103a and hsa-mir-494 during DENV-2 infection of endothelial HMEC-1 cell cultures. Our findings indicate that choosing an aligner would significantly impact the detection of the candidate sources of sfd-RNAs. Furthermore, re-inspecting expression profiles resulted in an accurate count of reads essential for differential expression analysis. Additionally, although the role of these sfd-RNAs during DENV infection remains to be validated, previously published findings provided strong evidence for potential cellular, molecular targets (CDK5 and PI3/AKT) of the miRNAs hsa-mir-103a and hsa-mir-494. The fact that tRFs are changed in various viral infections argues for including them in future studies.

## Methods

### Total RNA extraction and small RNA library preparation and sequencing

Cell lines and synchronized DENV2 infections are detailed in Alvarez-Diaz et al. (2019). DENV2-infected and uninfected HMEC-1 cells were considered for RNA extraction at four time points (3, 12, 24, and 48 h, each with three replicates). RNA of sizes ranging from 15 to 30 nt (small-sized fraction) and from 30 to 200 nt (medium-sized fraction) was extracted. A total of 48 libraries (24 for each size) were prepared and sequenced on the Illumina NextSeq 500 system by Exiqon facilities under the same protocol, as described in Alvarez-Diaz et al. (2019). The raw data of libraries are deposited at the NCBI, BioProject ID: PRJNA1018251.

### Small RNA-seq analysis

#### Consensus of multicopies of ncRNA loci

Sequences of miRNAs, tRNAs, and snoRNAs were obtained from UCSC (hg19), Rfam, snoRNABase download page, and tRNAscan-SE predictions. Subsequently, all sequences were mapped with BLASTN to the human genome (hg19) to search for multiple hits in the genome. Then, to define a unique set of homologous *loci*, the consensus of sequences was calculated using BLASTclust (Dondoshansky and Wolf, 2002) and bedtools (Quinlan and Hall, 2010). UCSC tracks included the miRBase version 21 (Griffiths-Jones et al., 2006) (a total of 1,871 pre-miRNAs), UCSC (a total of 939 pre-miRNAs) (Karolchik et al., 2004), and Rfam version 11.0 (a total of 1,229 pre-miRNAs) (Griffiths-Jones et al., 2005). Finally, we used 2,075 coordinates for 1,985 different miRNAs for further analysis. Following the same procedure, 937 snoRNA genomic *loci* were finally solved, starting from 858 genomic coordinates from Rfam and 486 from UCSC. Finally, previously detected human nuclear chromosomes of multi-sequences highly similar to human mitochondrial tRNAs (tRNA-lookalikes) (Telonis et al., 2015) increased the number of tRNA genomic positions to 1,129. Thus, we used the same procedure applied to miRNAs and snoRNAs to get a final set of 940 unique sequences for tRNAs used in further steps.

#### Raw data pre-processing and quality control

We evaluate the quality of the NGS data using FastQC (0.11.2). Low-quality reads with lengths less than 16 nt were dismissed. The over-representative 3’ adapters reported were also trimmed to avoid the high false-positive rates and detect typical clippers when applied to small RNA libraries.

#### Mapping of reads

Four aligners, namely, BWA (Li and Durbin, 2009), Bowtie (Langmead et al., 2009), Bowtie2 (Langmead and Salzberg, 2012), and segemehl (Hoffmann et al., 2009), were used to map reads to the human genome reference hg19. Tags were used to represent equal reads to reduce the mapping computational time.

Parameters used in this analysis are described in Tam et al. (2015). SAM outputs were converted to BAM format using SAMtools (Li et al., 2009). BAM files were used as the inputs to search for block expressions. Reads were considered a favorable map if they matched with the consensus ncRNAs on the same strand and inside the locus boundaries.

#### Searching for expression patterns and fragment discrimination in blocks

In the first step, blocks of mapped reads were calculated by Blockbuster (Langenberger et al., 2009). In the second step, the BAM format of mapped reads in consensus ncRNAs was filtered by size to filter the expected size of sfd-RNAs candidates (17–30 nt long). Then, a routine to tune reads assigned to block candidates to sfdRNAs was implemented (https://github.com/AimerGDiaz/NBlockTester). NBlockTester starts calculating the midpoint of each read *u* by *μ*_*u*_ = (*b*_*u*_ + *a*_*u*_)/2, where *a*_*u*_ and *b*_*u*_ correspond to the start and end positions, respectively, and variance $\sigma_{u}^{2}$ is defined by *σ*_*u*_ = (*b*_*u*_ − *a*_*u*_)/2. To avoid misclassified reads of sfd-RNAs in a block, we introduced a symmetry criterion to tune the classification of reads in blocks. Here, we started calculating the minimum midpoint from a set of midpoints, i.e., a set of reads; the initial symmetry range *sr* is defined by $sr=\mu_{u_{0}}+\sigma_{u_{0}}*1/2$ . Then, *μ* is calculated for the next read and compared with *sr*. If *μ* < *sr*, this read will be a candidate for this block. In summary, for all the reads clustered in a block in a candidate locus, the minimum midpoint value corresponds to the lowest mid coordinate of all the reads mapped to it. Then, for the growing block, the mean and the variance will be replaced by the arithmetic average $\hat{x}=\frac{1}{n}\sum_{i=1}^{n}x_{i}$ and the standard deviation $s=\sqrt{\frac{1}{n-1}\sum_{i=1}^{n}{\left(x_{i}-\hat{x}\right)}^{2}}$ , where *x*_*i*_ represent the starting and end coordinates $a_{u_{i}}$ , $b_{u_{i}}$ , $a_{u_{i+1}}$ , $b_{ui+1},\ldots ,a_{u_{n}}$ , $b_{u_{n}}$ i.e., calculated on each step with the accumulated reads of the growing block. The full explanation of the procedure is summarized in Figure 4.

#### Counting reads in blocks

Once blocks of read candidates to represent the expression of sfd-RNAs were set, we traced the reads to identify those mapped to multiple *loci*. Mapped reads from each ncRNA locus were retrieved with SAMtools using the coordinates of the ncRNA locus (independently of the annotation source). By the procedure explained in the *Consensus of multicopy of the ncRNA loci procedure*, those mapped reads were merged on consensus ncRNA categories. Then, to solve the MMR problem, a filter was applied to the predicted read blocks for each Bowtie and segemehl aligner, reducing identical multi-mapped reads to a unique representative read. However, in the case of the aligners Bowtie2 and BWA, which map pseudo-randomly on multiple homologous targets, the merged outcome will be used in the subsequent analysis.

### Discriminating between degradation fragments or non-fragmented ncRNAs and induced sfd-RNAs by dengue virus infection

Expression levels from the small- and medium-sized fractions were compared between infected and uninfected cell cultures at each time point described above. Then, to discard functional subproducts (putative sfd-RNAs) induced by dengue virus infection of degradation fragments or non-fragmented ncRNAs, we first compared and contrasted the basal expression of ncRNAs in uninfected (mock cells) with the expression of ncRNAs from infected cells using the medium-sized fraction. Then, in the second step, the small-sized fraction was used to detect differential expression by simultaneously contrasting the block expression against the controls in both fractions. So a block (putative sfd-RNAs) was declared differentially expressed if a change in read counts or expression levels was statistically found any time post-infection, but none in any control.

### Differential expression analysis

Differential expression analyses were performed in the R statistical environment (v3.2.0) using edgeR (v3.2.0) software (Bioconductor v2.12 repository). DENV2-infected and uninfected HMEC-1 cells at 3, 12, 24, and 48 h (with three replicates) were considered factors in constructing the design matrix used as input to estimate dispersions according to the Cox–Reid profile-adjusted likelihood (CR) method. We used four normalization methods, i.e., counts per million (CPM), upper quartile scaling (UQS), trimmed mean of M (TMM), and relative log expression (RLE), for the differential expression analyses following the indications of Tam et al. (2015). The read count in the design matrix was fitted to a generalized linear model (GLM). Fold changes greater than 1.0 or lower than −1.0 and FDR less than 0.05 were used as thresholds to select the top set of differentially expressed sfd-RNA candidates. Then, our approach, coupled with four aligners and four normalization methods, lets us define sixteen different computational strategies. Then, a rank order of prediction for each sfd-RNA is measured as 1 for the sixteen computational strategies that predicted it as differentially expressed. Coordinates of hg19 and accepted HGCN names of potential sources of sfd-RNAs, which were statistically significant, were updated to the Genome Reference Consortium Human GRCh38.p14 (GCA_000001405.29) and UCSC Genome Browser assembly ID: hg38 using bigBedToBed and LiftOver available at UCSC (consulted on 29 November 2023).

## Data Availability

The datasets presented in this study can be found in online repositories. The names of the repository/repositories and accession number(s) can be found below: (Bioproject Accession Number: PRJNA1018251).

## Supplementary material

SUPPLEMENTARY TABLE S1 Identified DE of sfd-RNA of (small-sized fraction) GRCh38.p14 coordinates and HGCN names.

SUPPLEMENTARY TABLE S2 Identified DE of sfd-RNA of (small-sized fraction) GRCh38.p14 coordinates and non-assigned HGCN names.

SUPPLEMENTARY TABLE S3 Identified DE of sfd-RNA of (medium-sized fraction) GRCh38.p14 coordinates and HGCN names.

SUPPLEMENTARY TABLE S4 Identified DE of sfd-RNA of (medium-sized fraction) GRCh38.p14 coordinates and non-assigned HGCN names.
