# Supplementary figures and images for: Systematic computational hunting for small RNAs derived from ncRNAs during dengue virus infection in endothelial HMEC-1 cells
Source: Front Bioinform. 2024 Jan 31;4:1293412. doi: 10.3389/fbinf.2024.1293412 (PMC10864640; doi:10.3389/fbinf.2024.1293412)

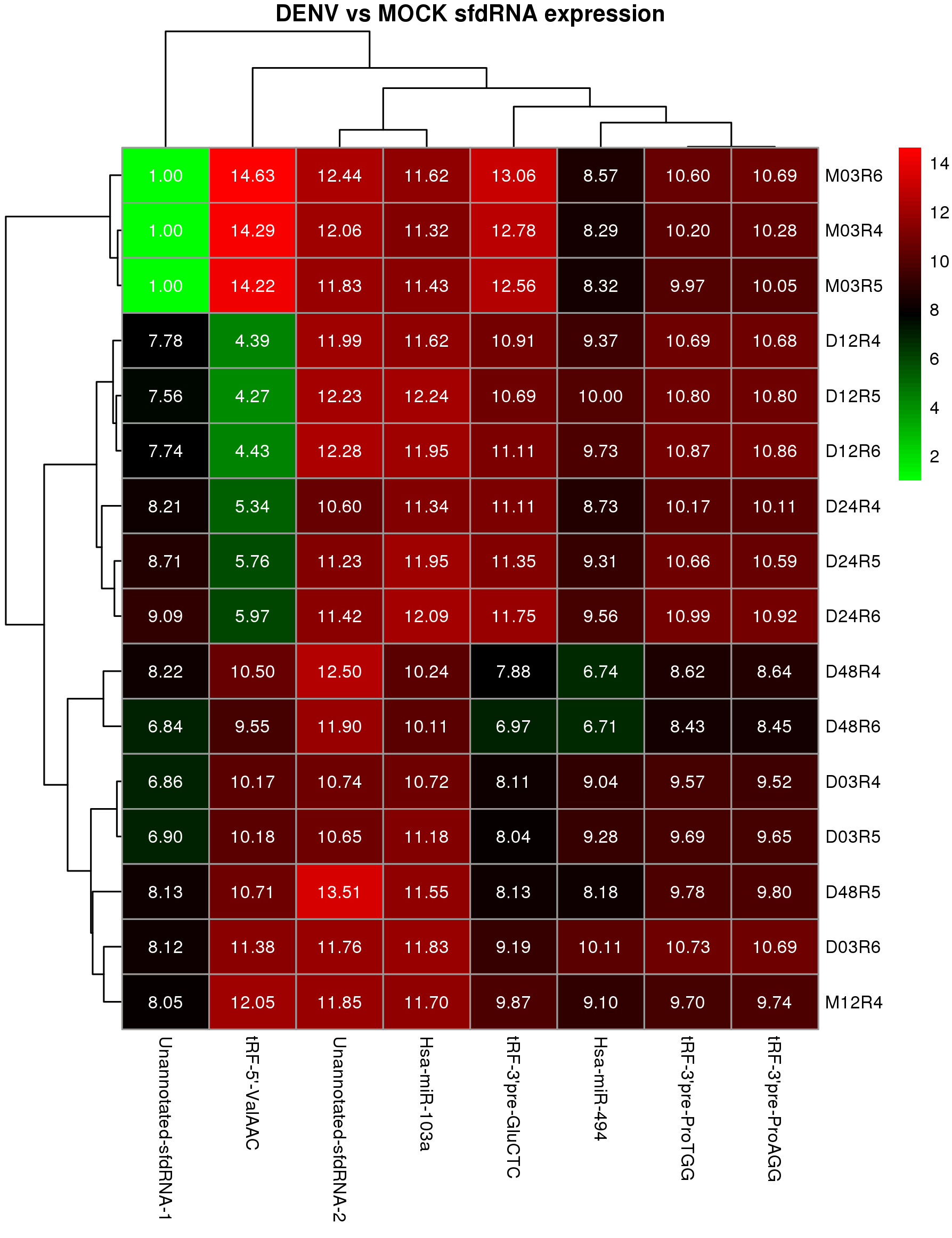

Supplement: Supplementary file 3 [file Image1.JPEG]
